# Calibrated BOLD using direct measurement of changes in venous oxygenation

**DOI:** 10.1016/j.neuroimage.2012.08.045

**Published:** 2012-11-15

**Authors:** Ian D. Driver, Emma L. Hall, Samuel J. Wharton, Susan E. Pritchard, Susan T. Francis, Penny A. Gowland

**Affiliations:** Sir Peter Mansfield Magnetic Resonance Centre, University of Nottingham, Nottingham, United Kingdom

**Keywords:** BOLD calibration, Hyperoxia, fMRI, CMRO_2_, Blood oxygenation

## Abstract

Calibration of the BOLD signal is potentially of great value in providing a closer measure of the underlying changes in brain function related to neuronal activity than the BOLD signal alone, but current approaches rely on an assumed relationship between cerebral blood volume (CBV) and cerebral blood flow (CBF). This is poorly characterised in humans and does not reflect the predominantly venous nature of BOLD contrast, whilst this relationship may vary across brain regions and depend on the structure of the local vascular bed. This work demonstrates a new approach to BOLD calibration which does not require an assumption about the relationship between cerebral blood volume and cerebral blood flow. This method involves repeating the same stimulus both at normoxia and hyperoxia, using hyperoxic BOLD contrast to estimate the relative changes in venous blood oxygenation and venous CBV. To do this the effect of hyperoxia on venous blood oxygenation has to be calculated, which requires an estimate of basal oxygen extraction fraction, and this can be estimated from the phase as an alternative to using a literature estimate. Additional measurement of the relative change in CBF, combined with the blood oxygenation change can be used to calculate the relative change in CMRO_2_ due to the stimulus. CMRO_2_ changes of 18 ± 8% in response to a motor task were measured without requiring the assumption of a CBV/CBF coupling relationship, and are in agreement with previous approaches.

## Highlights

► Venous blood oxygenation and CBV changes measured in response to a motor task. ► BOLD response to a motor task compared at normoxia and hyperoxia. ► BOLD calibration without assuming CBF/CBV coupling relationship. ► Motor task CMRO_2_ response of 18 ± 8% agrees with previous literature values.

## Introduction

Blood oxygenation level dependent (BOLD) contrast is widely used in functional magnetic resonance imaging (fMRI) to monitor brain function. However, the BOLD signal does not provide a direct measurement of brain function, but rather it monitors the haemodynamic response to changes in the underlying brain function. As a result the BOLD signal is blurred both spatially and temporally with respect to the underlying changes in neuronal function, and the amplitude of the BOLD signal has a complex, indirect relationship on the amplitude of the neuronal activity, which depends amongst other things on the local vascular structure and reactivity (Boynton et al., 1996; Buxton et al., 1998; Friston et al., 1998; Turner, 2002; Shmuel et al., 2007). These vascular confounds restrict the conclusions that can be drawn from a BOLD experiment, particularly in situations where either the baseline cerebral blood flow (CBF) and cerebral blood volume (CBV), or the capacity of the cerebral vasculature to respond may be altered, such as in pharmacological studies or some pathologies. Baseline and dynamic haemodynamic properties will combine, along with the cerebral metabolic rate of oxygen consumption (CMRO_2_), to determine the amplitude of a BOLD signal response to changes in brain function.

BOLD contrast depends primarily on changes in venous blood oxygenation (Y) and venous cerebral blood volume (vCBV) (Ogawa et al., 1993), and in turn, Y depends on CMRO_2_ and CBF, whilst the vCBV is thought to be coupled to CBF (Grubb et al., 1974; Buxton et al., 1998). Thus the BOLD signal depends on the changes in vCBV, CBF and CMRO_2_.

CMRO_2_ is closely related to tissue energy demand (Rothman et al., 1999), so is expected to provide a more direct measure of underlying brain function than the BOLD signal, as it is less affected by local haemodynamic properties. Task related changes in CMRO_2_ have previously been calculated by measuring BOLD and CBF changes in response to a task (Davis et al., 1998). This approach requires knowledge of a calibration parameter, which is the BOLD signal change that would be achieved if the venous blood volume were fully oxygenated. This calibration parameter can be calculated using measured BOLD and CBF changes in response to hypercapnia (Davis et al., 1998), or by using BOLD changes in response to hyperoxia combined with a model estimating the effect of hyperoxia on venous blood oxygenation (Chiarelli et al., 2007a).

Regardless of whether hypercapnia or hyperoxia is used, this technique assumes that CBV is coupled to CBF for steady state conditions, instead of directly measuring CBV. This coupling relationship was initially measured in an animal model (Grubb et al., 1974) as a power law relationship, such that

(1) $$\text{CBV}\propto {\text{CBF}}^{\alpha }\text{,}$$

where  *α* = 0.38. Results subsequently obtained in humans suggested that *α* = 0.29 (Ito et al., 2003). However, this relationship refers to the total CBV, whereas the BOLD signal arises primarily from the vascular component containing deoxygenated haemoglobin (dHb) (i.e. vCBV corresponding to veins and venules). Furthermore, vCBV has been shown to increase relatively less than total CBV, in response to both hypercapnia (Lee et al., 2001) and forepaw stimulation (Kim et al., 2007) in rats. The coupling relationship between vCBV and CBF has been measured in humans as *α* = 0.23 during a combined motor and visual task (Chen and Pike, 2009) and *α* = 0.18 in response to hypercapnia (Chen and Pike, 2010), indicating that using the total CBV rather than vCBV coupling relationship for calibrated BOLD will lead to an underestimation of CMRO_2_ (Chen and Pike, 2009). Nonetheless the coupling between vCBV and CBF remains poorly characterised in humans, and may vary with brain region and in pathology.

In this work we propose a new approach to BOLD calibration, using BOLD contrast on hyperoxia and normoxia, to calculate the relative change in venous CBV and Y due to the stimulus, avoiding the need to make any assumption about the coupling between vCBV and CBF.

## Theory

Hyperoxia causes an increase in cerebral venous blood oxygenation (Yv), providing a BOLD contrast which has previously been used to measure both absolute resting vCBV (Bulte et al., 2007a) and the fractional change in vCBV in response to a task (Blockley et al., 2012). It will be shown that if the same task is performed at both normoxia and hyperoxia then the relative change in Yv due to the task can also be measured, assuming that hyperoxia has no effect on either CBF or CMRO_2_. If the change in CBF due to the task is also measured, then the relative change in CMRO_2_ (rCMRO_2_) due to the task can be calculated (Davis et al., 1998) without assuming the relationship between vCBV and CBF.

Table 1 lists the parameters used in the following description of the method.

### Model for calculating relative task-related CMRO_2_ changes

An analytical model is used to describe the effect of hyperoxia on tissue *R*_2_*, (Yablonskiy and Haacke, 1994).

(2) $$R_{2}^{*}=kV\left(1-\text{Y}\right)+R_{2,0}^{*}$$

where *R*_2,0_* is the transverse relaxation rate of tissue containing only fully oxygenated blood vessels, Y is the blood haemoglobin oxygen saturation fraction and *V* is the volume fraction occupied by blood vessels. The term *k* is a constant arising from an extravascular signal model, based on the static dephasing regime of spins located around randomly orientated blood vessels:

(3) $$k=\frac{4}{3}\pi \text{Δ}\chi \left[{\text{Hb}}_{\text{tot}}\right]B_{0}$$

where the 4*π*/3 term describes the effect of vessels with random orientation, Δ*χ* is the susceptibility of deoxygenated haemoglobin relative to tissue, [Hb_tot_] is the total haemoglobin concentration and *B*_0_ is the static magnetic field. This model assumes a linear relationship between *R*_2_* and deoxygenated haemoglobin fraction (β = 1 in Davis model (Davis et al., 1998)) which is more appropriate for 7 T (Yablonskiy and Haacke, 1994; Driver et al., 2010; Croal et al., 2012a).

The venous dHb fraction  *Q* is defined such that *Q* = (1 − Yv) and *Q*_0_ denotes the normoxic rest condition (which is also equivalent to oxygen extraction fraction (OEF)). Hyperoxia increases venous oxygenation Yv, such that on hyperoxia  *Q* = (*Q*_0_ + Δ*Q*_h_) = *Q*_0_(1 + q_h_), where Δ*Q*_h_ is the absolute change in venous dHb fraction due to hyperoxia and q_h_ is the relative change in venous dHb due to hyperoxia. Note that since hyperoxia will cause an increase in Yv, *ΔQ*_h_ and q_h_ will be negative. An analogous term q_act_ can be defined for the relative change in *Q* due to task related changes in CMRO_2_ and CBF. Therefore, the venous dHb fraction during a task performed at hyperoxia is *Q* = (*Q*_0_ + Δ*Q*_h_ + Δ*Q*_act_) = *Q*_0_(1 + q_h_ + q_act_), assuming that the amount of oxygen extracted by the capillary bed is independent of hyperoxia at steady state (i.e. q_act_ is the same at normoxia and hyperoxia).

**Fig. 1:**
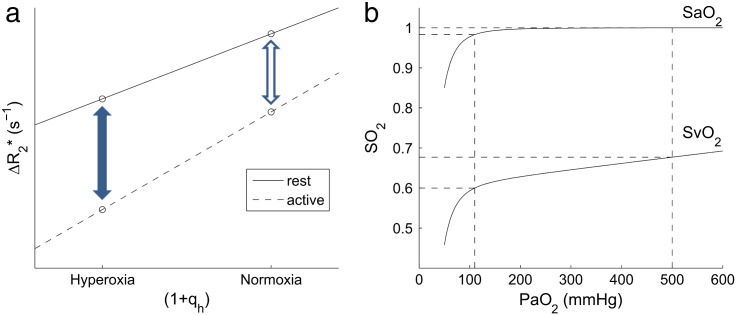
(a) A schematic diagram of *R*_2,rest_* and *R*_2,act_* plotted against venous blood oxygenation, indicating the BOLD signal change on normoxia (open arrow) and hyperoxia (solid arrow). The linear fits shown are used to calculate rvCBV and q_act_ as described in the Theory section. (b) Simulated relationship between SaO_2_ and SvO_2_ (= 1 − *Q*) and PaO_2_. The broken lines indicate arterial and venous oxygen saturation for 110 mm Hg (~ 21% O_2_, normoxia) and 500 mm Hg (~ 60% O_2_). Values used in this simulation are PaO_2,0_ = 110 mm Hg, OEF = 0.4, *ϕ* = 1.34 ml(O_2_)/g, [Hb] = 15 g/dl_blood_ and *ε* = 0.0031 ml/(dl_blood_·mm Hg).

The transverse relaxation rates during the neuronal activation *R*_2,act_* and rest *R*_2,rest_* at a given level of hyperoxia (q_h_), can be modelled as

(4) $$R_{2,\text{rest}}^{*}\left({\text{q}}_{\text{h}}\right)=kV_{0}Q_{0}\left(1+{\text{q}}_{\text{h}}\right)+R_{2,0}^{*}$$

(5) $$\begin{array}{c} R_{2,\text{act}}^{*}\left({\text{q}}_{\text{h}}\right)=k\left(V_{0}+\text{Δ}V_{\text{act}}\right)Q_{0}\left(1+{\text{q}}_{\text{h}}+{\text{q}}_{\text{act}}\right)+R_{2,0}^{*}\\ =k\left(V_{0}+\text{Δ}V_{\text{act}}\right)Q_{0}\left(1+{\text{q}}_{\text{h}}\right)+k\left(V_{0}+\text{Δ}V_{\text{act}}\right)Q_{0}{\text{q}}_{\text{act}}+R_{2,0}^{*} \end{array}$$

where *V*_0_ is the resting vCBV and Δ*V*_act_ is the absolute change in venous CBV due to neuronal activation. *R*_2,0_* is the transverse relaxation rate of the tissue neglecting any venous effects, and includes effects of shimming. The method for estimating q_h_ is discussed in Estimating qh theory section. If *R*_2,act_* and *R*_2,rest_* are measured for two or more values of q_h_ then the gradient of a plot of *R*_2,rest_* versus (1 + q_h_) (Fig. 1a) will be *kV*_0_*Q*_0_, which is the calibration constant M used in previous calibrated BOLD experiments (Davis et al., 1998; Chiarelli et al., 2007a). The gradient of the plot of *R*_2,act_* versus (1 + q_h_) (Fig. 1a) is *k*(*V*_0_ + Δ*V*_act_)*Q*_0_, which will be referred to as M′. The difference in intercepts of the rest and activation curves is *k*(*V*_0_ + Δ*V*_act_)*Q*_0_q_act_, which can be divided by M′ to give q_act_. The ratio of the activation to rest gradients gives the relative venous CBV (rvCBV) change on activation:

(6) $$\frac{\text{M}\prime }{\text{M}}=\left(1+\frac{\text{Δ}V_{\text{act}}}{V_{0}}\right)=\left(1+\text{rvCBV}\right)\text{.}$$

If the relative change in CBF (rCBF) on activation can be measured (e.g. using arterial spin labelling (ASL)), then Fick's principle can be combined with the value of q_act_ to estimate the relative change in CMRO_2_ (rCMRO_2_):

(7) $$\left(1+{\text{rCMRO}}_{2}\right)=\left(1+{\text{q}}_{\text{act}}\right)\left(1+\text{rCBF}\right)\text{.}$$

To summarise, this method does not assume a coupling relationship between CBV and CBF (Grubb et al., 1974), but does assume negligible vasoconstriction due to hyperoxia (which is reasonable in the case of normocapnic hyperoxia (Croal et al., 2012b)) and that oxygen consumption is independent of hyperoxia so that q_act_ is the same at normoxia and hyperoxia (an area that needs further investigation). This approach also assumes that the haematocrit remains constant both during hyperoxia and the motor task. Furthermore to create the plots described above, q_h_, the relative change in venous dHb due to hyperoxia, must be known; a method for determining q_h_ is described in the next section.

### Estimating q_h_

During hyperoxic periods, arterial oxygen partial pressure (PaO_2_) is increased, with most of this extra oxygen being dissolved in arterial plasma, since arterial haemoglobin is already close to being fully saturated at normoxia. Oxygen dissociation in blood is described by a widely accepted model (Severinghaus, 1979). At 310 K and a pH of 7.4, the relationship between arterial haemoglobin oxygen saturation (SaO_2_) and PaO_2_ is

(8) $${\text{SaO}}_{2}={\left(\frac{23,400}{{{\text{PaO}}_{2}}^{3}+150{\text{PaO}}_{2}}-1\right)}^{-1}\text{,}$$

plotted in Fig. 1b. Here end-tidal PO_2_ (P_ET_O_2_) was used to estimate PaO_2_ (i.e. P_ET_O_2_ = PaO_2_) assuming that arterial blood gases are in equilibrium with alveolar gas. This assumption is not true in general as there are partial pressure gradients for oxygen between exhaled gas and alveolar gas due to non-uniform alveolar distribution of inhaled gas, and between alveolar gas and arterial blood due to the non-uniform distribution of lung perfusion (Ayres et al., 1964). The sum of these effects results in end-tidal to arterial gradients of less than 50 mm Hg in healthy subjects (Ayres et al., 1964). This PO_2_ difference at hyperoxia would correspond to an error in calculation of plasma O_2_ content of 0.15 ml per decilitre, or less than 0.7% of total blood O_2_ content which would have a negligible effect on the calculation of q_h_. However in our study, end-tidal to arterial PO_2_ gradient was reduced further by several factors unique to our method of controlling the PaO_2_ (see Respiratory paradigm section). The employment of the sequential gas delivery circuit to administer the O_2_ (in which gas in equilibrium with alveolar blood gas occupies the anatomical dead space) resulted in a more homogeneous distribution in alveolar PO_2_ (Swenson et al., 1994; Brogan et al., 2004; Ito et al., 2008). This minimises the gradient between expired and alveolar gas, and also eliminates the effect of the variability of distribution of pulmonary blood flow to PaO_2_ (Ito et al., 2008; Fierstra et al., 2011).

The total arterial blood oxygen content (CaO_2_) is the sum of the oxygen bound to haemoglobin and that dissolved in plasma

(9) $${\text{CaO}}_{2}=\left(\phi \cdot \left[\text{Hb}\right]\cdot {\text{SaO}}_{2}\right)+\varepsilon \cdot {\text{PaO}}_{2}$$

where *ϕ* = 1.34 ml(O_2_)/g is the oxygen carrying capacity of haemoglobin, [Hb] = 15 g/dl_blood_ is the concentration of haemoglobin and *ε* = 0.0031 ml/(dl_blood_·mm Hg) is the solubility coefficient of oxygen in blood (Chiarelli et al., 2007a). Dissolved oxygen meets part of the tissue oxygen requirement (Rostrup et al., 1995).

Venous haemoglobin oxygen saturation (SvO_2_) can be estimated using the method proposed by Chiarelli et al. (2007a). The total venous blood oxygen content (CvO_2_) is what remains after oxygen has been extracted from capillaries, and is given by

(10) $${\text{CvO}}_{2}={\text{CaO}}_{2}-\text{OE}$$

where OE is the oxygen extraction. OE is assumed to be independent of the level of hyperoxia, and OE = CaO_2,0_ ⋅ OEF, where CaO_2,0_ is the total arterial oxygen content at normoxia and OEF is the oxygen extraction fraction at normoxia (= *Q*_0_). Since venous haemoglobin is not close to being fully saturated under normobaric hyperoxia, it can be assumed that a negligible amount of the CvO_2_ is carried as dissolved oxygen. This means that Eqs. (9) and (10) can be combined to give venous oxygen saturation:

(11) $$\left(1-Q\right)={\text{CvO}}_{2}/\left(\phi \cdot \left[\text{Hb}\right]\right)=\frac{\left(\phi \cdot \left[\text{Hb}\right]\cdot {\text{SaO}}_{2}\right)+\varepsilon \cdot {\text{PaO}}_{2}-\text{OE}}{\left(\phi \cdot \left[\text{Hb}\right]\right)}\text{,}$$

which is also plotted in Fig. 1b. This model can be used to estimate (1 − *Q*) from PaO_2_, which can be estimated by measuring exhaled gas oxygen fraction.

Since OE is assumed to be independent of hyperoxia level, Δ*Q*_h_ can be estimated independently of OE or OEF, but *Q*_0_ must be estimated. There are two possible ways of doing this and we have attempted both in this paper. First the value can be taken from literature values of OEF, (we used 0.4 here). Alternatively, in principle it can be measured from venous blood (e.g. from the T_2_ (Lu and Ge, 2008) or susceptibility as used here (Jain et al., 2010), see Analysis section).

## Materials and methods

Nine healthy volunteers (3 male, 6 female; mean age = 27 ± 3 years, range 23–30 years) participated in this study. Ethical approval was given by the University of Nottingham Medical School Ethics Committee and all subjects gave informed written consent prior to participating. For one subject there was a significant change in P_ET_CO_2_ (> 1 mm Hg) during the transitions into both hyperoxia periods, which overlapped with the motor trials at hyperoxia and so this subject was discarded from further analysis.

### Motor paradigm

All subjects were asked to perform a motor task during a respiratory challenge, so that the same motor task was repeated both at normoxia and hyperoxia. The motor task consisted of a bilateral sequential finger tap. This was visually cued, with the word ‘TAP’ displayed in red on a dark background on a projector screen at the end of the magnet bore. This was replaced by a white ‘+’ fixation point during rest periods.

**Fig. 2:**
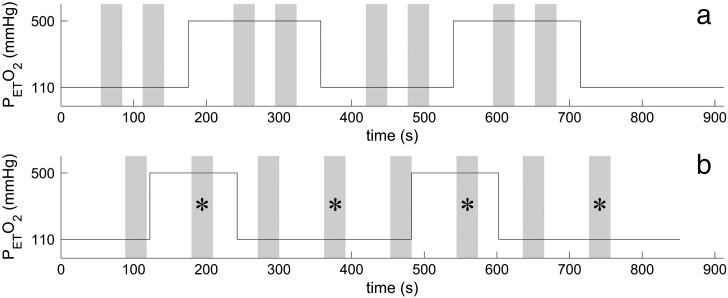
An illustration of the combined hyperoxia and motor task for (A) the 30 s ON/30 s OFF paradigm (Paradigm A) and (B) the 30 s ON/60 s OFF paradigm (Paradigm B). The motor task is shown in grey. Trials marked with an * were used in the analysis for (B).

The respiratory challenge consisted of two repeats of 3 min of normoxia, followed by 3 min of hyperoxia, with a final 3 min of normoxia at the end. The motor paradigm consisted of blocks of two trials of 30 s ON/30 s OFF at each gas level, with the block finishing just before the start of a transition between the two gas levels (see Fig. 2A — Paradigm A). For the final subject, the paradigm was modified to maintain the subject's attention better: evenly spaced motor trials of 90 s of rest followed by 30 s ON/60 s OFF were superimposed on 2 min of normoxia, followed by two repeats of 2 min hyperoxia and 4 min of normoxia (see Fig. 2B — Paradigm B). Alternate trials fell at a respiratory transition and could not be used, giving 4 usable trials in total.

The last 4 subjects (indicated in Table 3) were also asked to complete an additional motor task during an ASL acquisition to allow the relative change in CBF to be measured, and thus the relative change in CMRO_2_ to be estimated. This task consisted of 10 trials of Paradigm A or 8 trials of Paradigm B, performed with the subject breathing medical air.

### Respiratory paradigm

A feed-forward, low gas flow system (RespirAct™, Thornhill Research Inc., Toronto, Canada) and a sequential gas delivery (SGD) breathing circuit (Banzett et al., 2000; Slessarev et al., 2007) were used to target end-tidal PCO_2_ (P_ET_CO_2_) and PO_2_ (P_ET_O_2_) independently (Slessarev et al., 2007). Source gases used by the system were O_2_, air, and two gas blends of N_2_, CO_2_ and O_2_, so that all source gases contained safe concentrations of O_2_. The RespirAct™ follows the approach of Slessarev et al. (2007) to calculate the required flows of these source gases into the SGD breathing circuit to attain the targeted P_ET_CO_2_ and P_ET_O_2_ values. For the normoxic condition, both P_ET_O_2_ and P_ET_CO_2_ were maintained at the subject's resting values (~ 110 mm Hg and ~ 40 mm Hg, respectively). For the hyperoxic condition, P_ET_O_2_ was targeted at 500 mm Hg, whilst P_ET_CO_2_ was maintained at the resting value.

### Data acquisition

Scanning was performed on a Philips Achieva 7 T system, with head volume transmit and 16 channel SENSE head receive coil. Gradient echo (GE) EPI data were acquired every 2.4 s throughout the respiratory and motor tasks. Images consisted of 2 mm isotropic voxels, with a 192 × 192 mm^2^ field-of-view, and 20 axial slices (2 mm thickness, no slice gap) spanning the motor cortex. Imaging parameters were TE = 25 ms, SENSE factor = 3, voxel band-width = 41.5 Hz, TR = 2.4 s and flip angle = 75°. ASL datasets were acquired using a FAIR labelling scheme, with TI = 1400 ms, selective thickness 10 mm wider than the imaging volume, non-selective thickness of 300 mm and background suppression pulses at 402 and 639 ms (Garcia et al., 2005); in-plane pre- and post-saturation were used. These images consisted of 2 × 2 × 4 mm^3^ voxels, with a 192 × 192 mm^2^ field-of-view, and 8 axial slices (4 mm thickness, no slice gap) with the same orientation and centre as the GE EPI data. Imaging parameters were TE = 14 ms, SENSE factor = 3, voxel band-width = 41.5 Hz, TR = 3 s (6 s for a tag/control pair). Two equilibrium magnetisation images were acquired for signal normalisation (same parameters as for the ASL, except with no inversion and a long TR of 10 s), one before and one after the ASL acquisition.

### Analysis

GE EPI datasets were motion corrected using MCFLIRT (FSL, fMRIB, Oxford, UK). Voxelwise linear detrending was then performed using a linear fit (MATLAB, The MathWorks, Natick, USA) to baseline timepoints (i.e. during periods of normoxia and not within 30 s of a previous finger tap). Linear detrending was chosen in preference to a high-pass temporal filter, due to the long cycle lengths of the paradigm. Maps of statistical significance of the BOLD response to the motor task were formed using the FEAT (FSL, fMRIB, Oxford, UK) general linear model. This was done on the datasets before voxelwise normalisation, but after motion correction and linear detrending. A boxcar design, representing the hyperoxia response was included in the model design as an independent regressor, so the hyperoxia-based BOLD response could be separated from the motor response in the analysis. ‘BOLD motor activation masks’ were formed of clusters of voxels with Z > 5 and P_cluster_ < 0.05 for the response to the motor task.

Since the ASL data was background suppressed, this made motion correction problematic due to the low signal of the tag and control images. Instead the two equilibrium magnetisation images from the start and end of the ASL data set were subtracted to determine whether there was any systematic motion through the dataset, and no more than 1 voxel of displacement was found across all subjects. The interleaved acquisition of tag and control ASL images meant that linear temporal interpolation (MATLAB, The MathWorks, Natick, USA) had to be performed on both the tag and control datasets. ASL subtraction was then performed between interpolated tag and control datasets, resulting in a CBF-weighted timecourse with timepoints every 3 s, but a real temporal resolution of 6 s. Maps of statistical significance of the CBF response to the motor task were formed using the FEAT (FSL, fMRIB, Oxford, UK) general linear model. In this analysis, a high-pass filter was used with a cut-off period of twice the trial length, corresponding to 120 s for Paradigm A and 180 s for Paradigm B. ‘CBF motor activation masks’ were formed of clusters of voxels with Z > 2.3 and P_cluster_ < 0.05. The lower Z-statistic threshold was used due to the intrinsically lower SNR of ASL compared with GE EPI BOLD.

**Fig. 3:**
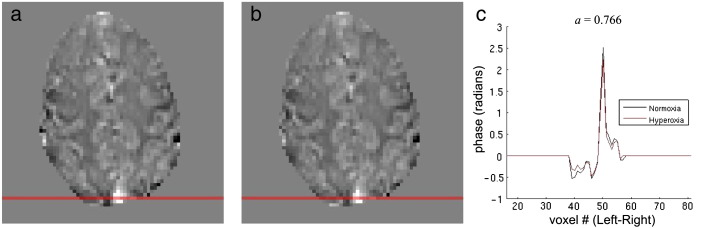
Example data illustrating the phase-based calculation of *Q*_0_. Average (a) normoxia and (b) hyperoxia phase maps (range − 1 to 1 rad), with the selected line profile position highlighted in red. (c) Line profiles for normoxia (black) and hyperoxia (red). The fitted ratio *a* of hypercapnia phase to normocapnia phase is shown for this subject.

Timecourses of measured P_ET_O_2_ were used to estimate the absolute change in venous deoxyhaemoglobin fraction due to hyperoxia (Δ*Q*_h_) (see Theory section). *Q*_0_ and hence the relative change (q_h_) was then calculated using an assumed resting OEF of 0.4. In addition, in an attempt to avoid the need to assume resting OEF, phase data constructed from the GE EPI dataset were used to calculate an alternative value of *Q*_0_ and hence q_h_. The phase data were unwrapped in space over each volume (PRELUDE, FSL) and then unwrapped over time (UNWRAP, MATLAB). The motion correction transformations from the magnitude data were applied to the unwrapped phase data (MCFLIRT, FSL). To remove the global phase shift caused by increased oxygen concentrations in the frontal sinus and nasal cavity (Driver et al., 2011; Pilkinton et al., 2011), the phase data were high-passed filtered using a homodyne filter with a smoothing kernel with a FWHM of 4 mm (Hammond et al., 2008). The filtered phase data were then separated into normoxia and hyperoxia periods, defined as the final 2 min of each period (1 min for paradigm B). A line profile was taken across the sagittal sinus, to estimate the extravascular phase shift due to dHb in the sagittal sinus both at normoxia and hyperoxia (example in Fig. 3). A ratio of hyperoxia:normoxia phase (*a*) was calculated by a least-squares fit of the line profiles to the equation *ϕ*_hyperoxia_ = *a*·*ϕ*_normoxia_. Voxels with a large intravascular component (such as sagittal sinus) were excluded from the fit, by applying a high intensity threshold to the magnitude data, averaged over the normoxia period. Assuming that the susceptibilities of tissue and blood plasma are equal to that of water and that *a* is also equal to the ratio of hyperoxia:normoxia susceptibilities, relative to tissue; the following equation can be used to relate *a* and Δ*Q*_h_ to *Q*_0_ (see Appendix A):

(12) $$\left(1-Q_{0}\right)=\frac{\frac{\text{Δ}Q_{\text{h}}\cdot \left(\text{Δ}\chi_{\text{oxy}}-\Delta \chi_{\text{deoxy}}\right)}{\left(1-a\right)}-\text{Δ}\chi_{\text{deoxy}}}{\left(\text{Δ}\chi_{\text{oxy}}-\Delta \chi_{\text{deoxy}}\right)}$$

where Δ*χ*_oxy_ = − 0.017 × 10^− 6^, Δ*χ*_deoxy_ = + 0.247 × 10^− 6^ (Spees et al., 2001) are the volume susceptibilities of oxygenated haemoglobin and deoxygenated haemoglobin relative to water, respectively.

For the GE EPI datasets each voxel's timecourse was normalised by dividing by the average value over all baseline timepoints and then average timecourses were calculated over all voxels within the ‘BOLD motor activation mask’. In some cases, transient changes in P_ET_CO_2_ occurred during a P_ET_O_2_ transition, which persisted into the start of the next motor trial. Any motor trial which included a change in P_ET_CO_2_ of greater than 1 mm Hg compared to the normocapnia value was discarded. The average signal during rest and active periods (motor task) were compared to the normoxic baseline (defined earlier), to estimate rest and an active % signal change (%BOLD) for each trial and each hyperoxia/normoxia condition. These % changes were converted to Δ*R*_2_* changes using the approximation Δ*R*_2_* ≈ − %BOLD/(100TE), to estimate q_act_. Linear regressions (MATLAB, The MathWorks, Natick, USA) were performed to fit Δ*R*_2_* as a linear function of q_h_, separately for the rest and active conditions. The fitted parameters were then used to estimate q_act_ and rvCBV, as proposed in the Theory section.

For each of the four subjects for which ASL data were acquired an additional ‘combined mask’ was formed from the intersection of the ‘BOLD motor activation mask’ and ‘CBF motor activation mask’. The CBF-weighted data (after interpolation and subtraction) were normalised to the rest periods, by dividing by the average of the last half of all rest periods, and an average timecourse was calculated over all voxels in the ‘combined mask’. The % change in CBF on activation was calculated by averaging over the active (motor task) period of all trials, multiplied by 100. Values of q_act_ were recalculated for the ‘combined mask’ using the same method as described above, rCBF and q_act_ were used to calculate rCMRO_2_, using Eq. (7). Maps of the pixel values of M, rvCBV and q_act_ were also calculated.

## Results

Transitions in ΔP_ET_O_2_ of 330 ± 20 mm Hg (mean ± SEM over subjects) were achieved, which corresponded to an increase of ΔYv = 0.068 ± 0.003 and an 11% increase over the resting normoxia value. The average change in end tidal CO_2_ on hyperoxia compared to normoxia was ΔP_ET_CO_2_ = − 0.5 ± 0.1 mm Hg (range of − 1.1–0 mm Hg across subjects) which was assumed to be too small to have a significant effect on CBF and the resulting BOLD signal.

**Fig. 4:**
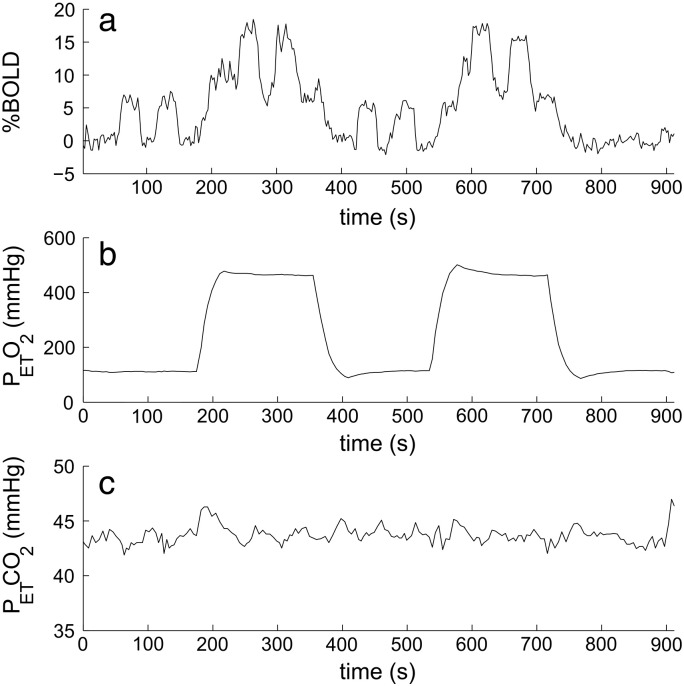
Example timecourses from a single subject for (a) BOLD, (b) P_ET_O_2_ and (c) P_ET_CO_2_ (BOLD motor activation mask).

**Fig. 5:**
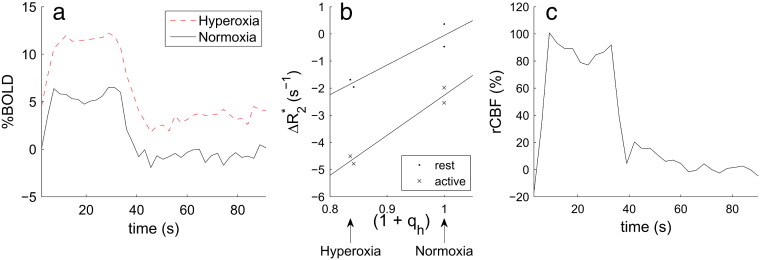
(a) Average motor trial %BOLD timecourses during hyperoxia (red dashed line) and normoxia (black line). (b) Example of the linear fit between *R*_2_* and (1 + q_h_). (c) The %CBF response to the motor task, averaged over trials. Data from subject 8, formed from the ‘combined mask’ (the intersection of BOLD and CBF motor activation masks).

Fig. 4 shows example BOLD, P_ET_O_2_ and P_ET_CO_2_ timecourses for subject 3 in the ‘BOLD motor activation mask’. Averaged across all subjects, the baseline BOLD signal increased by 6.4 ± 0.9% with hyperoxia and the BOLD response to the motor task was increased by 37 ± 7% on hyperoxia compared to normoxia. An example of an average trial of the motor task at normoxia and hyperoxia, as well as an example of the linear fits are shown in Fig. 5. The gradients of the linear fits gave M = 36 ± 5% and M′ = 48 ± 7%. Individual subject values for the relative increase in vCBV, relative decrease in deoxyhaemoglobin fraction q_act_ and corresponding absolute increase in Δ*Y*_*act*_ (= − *Q*_0_· q_act_) during the motor task are reported in Table 2. Monte Carlo simulations were performed to estimate the precision of the rvCBV and q_act_ values; 10,000 timecourses were simulated with the same temporal SNR (161) as the actual data and the resulting absolute standard deviation in the estimated values was *σ*(rvCBV) = 4.3 % and *σ*(q_act_) = 2.1 % with no significant systematic error.

An example CBF average trial timecourse is shown in Fig. 5c. Results based on the combined mask, rCBF, q_act_ and rCMRO_2_ are reported in Table 3, for both an assumed *Q*_0_ value of 0.4 and for *Q*_0_ calculated from the phase data. For the ‘combined mask’, values of M and M′ were calculated as M = 28 ± 2% and M′ = 34 ± 2%, and rvCBV = 22 ± 7% was lower in all four subjects compared with those values calculated from the ‘BOLD motor activation mask’.

**Fig. 6:**
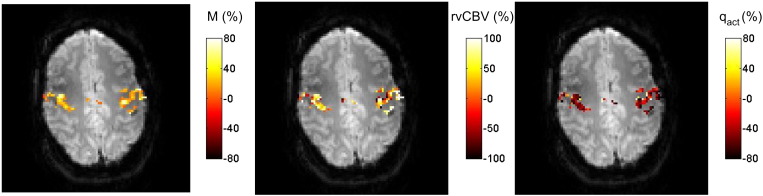
Voxelwise maps of M, rvCBV and q_act_ across the combined mask (the intersection of BOLD and CBF motor activation masks).

Voxel-by-voxel maps of M, rvCBV and q_act_ were formed by performing the above analysis on each voxel in the ‘combined mask’ and are shown in Fig. 6.

## Discussion

This paper has described a new method of using hyperoxia to measure CMRO_2_. By comparing the response to a task at hyperoxia and normoxia it provides an extra degree of information on the vascular nature of the BOLD signal. As a result, this method does not make any assumption about the coupling between CBV and CBF, but instead provides a direct measurement of the change in blood volume and oxygenation on activation with relatively low sensitivity to noise as indicated by the Monte Carlo simulations. Essentially this method is equivalent to the previous method proposed for hyperoxia based measurement of CMRO_2_ (Chiarelli et al., 2007a) except that instead of using the Grubb relationship, here rvCBV is measured directly (Blockley et al., 2012). Recently, hypercapnia and hyperoxia calibrated BOLD approaches have been combined to map basal OEF (Bulte et al., 2012; Gauthier and Hoge, 2012), relying on an assumed coupling relationship between CBV and CBF. These basal measurements could be extended by including a hypercapnia challenge repeated both at normoxia and hyperoxia to overcome the need to assume this relationship.

The rCMRO_2_ changes measured here agree with measurements made using hypercapnia-based calibration for a bilateral finger tap, with rCMRO_2_ = 16 ± 9 % (Kastrup et al., 2002) and 5–45 % (Chiarelli et al., 2007b), and for a right-handed finger tap using positron emission tomography giving rCMRO_2_ = 11 ± 13 % (Ito et al., 2005). The value of rvCBV = 22 ± 7% measured here is approximately twice that measured for a motor task by Chen and Pike (2009). Possible reasons for this difference could be due to differences in task performance, the region selected or the methods themselves, and it would be interesting to compare the two methods directly in future work. In this study there was a trend for a smaller increase in rvCBV in the ‘combined mask’ defined by both BOLD and CBF activated regions, compared with the ‘BOLD mask’. This suggests a smaller change in rvCBV in the microvasculature, compared with draining veins that contribute to the ‘BOLD mask’ but probably do not represent active tissue (Leontiev et al., 2007). The ‘combined mask’ was used for the estimate of CMRO_2_, to help to meet the extravascular signal assumption, by excluding large draining veins, whilst focusing the ROI on areas of active tissue.

This paper reports the first measurements of M in humans at 7 T. Equivalent values measured in the motor cortex are M ≈ 4 − 6 % at 3 T using hyperoxia or hypercapnia (Chiarelli et al., 2007a; Chiarelli et al., 2007b; Mark et al., 2011) and M ≈ 5 − 9 % at 1.5 T using hypercapnia (Kastrup et al., 2002; Stefanovic et al., 2004, 2005, 2006). An alternative approach at 3 T, calculating M by attempting to fully saturate venous blood using combined hypercapnia and hyperoxia (10% CO_2_ carbogen) reported M = 7.5–9.5% in the visual cortex (Gauthier et al., 2011). The increased value reported here (M = 36 ± 5 %, 8 subjects for BOLD activated region) is consistent with the increased BOLD contrast at 7 T and a smaller voxel volume causing less partial voluming. The ‘combined mask’ giving the region activated in both BOLD and CBF gave a lower value (M = 28 ± 2 %, 4 subjects), which would be expected due to the exclusion of large draining veins from this region.

All previous calibrated BOLD experiments have assumed a coupling relationship between CBV and CBF in Eq. (1), most using the relationship that was originally measured in rhesus monkeys, using PET and hypercapnia (*α* = 0.38) (Grubb et al., 1974). However since deoxygenated haemoglobin is the source of BOLD contrast, the venous, rather than total blood volume compartment should be used in the calibrated BOLD model. The venous compartment reacts passively to flow changes, whereas the arterial compartment actively constricts and dilates and so the coupling relationships for these two compartments would be expected to be different. Furthermore the global vascular response to a hypercapnic challenge may be different to the local response to neuronal activity, and the coupling relationship could change in pathology. Despite recent improvements in the understanding of this coupling (Lee et al., 2001; Kim et al., 2007; Chen and Pike, 2009, 2010), it remains preferable to avoid use of an assumed coupling constant.

Estimating q_h_ from P_ET_O_2_ requires an assumed value of *Q*_0_ which is equivalent to OEF (0.4 used here; see Eq. (10) and central columns on Table 3). OEF is thought to be fairly constant across healthy subjects, but it may vary in patients. The estimate of OEF does not affect the estimated change in absolute venous blood oxygenation, Δ*Q*_h_, but it will bias the relative change q_h_, since this is normalised to OEF. The calculation of rvCBV is independent of errors in OEF, but a systematic error in q_act_ will occur, which will be linearly proportional to the error in OEF, and this problem is common to all hyperoxia based methods of estimating rCMRO_2_ that use a literature value for OEF. An alternative approach is to estimate global OEF from venous blood oxygenation (Lu and Ge, 2008; Jain et al., 2010). We attempted this post hoc analysis here, using the change in blood susceptibility on hyperoxia to provide a method of estimating the baseline susceptibility in the vessel independent of any model of the pattern of the field shift around the vessel. The variability in measured *Q*_0_ (equivalent to OEF) across subjects from this technique is most probably dominated by errors in the technique, such as line profile selection and the underlying quality of the phase image, rather than biological inter-subject variability. Higher resolution gradient echo images and alternative data analysis approaches such as 2D rather than 1D profiles around the vessel, are likely to provide more reliable estimates of *Q*_0_. Although OEF is thought to be homogeneous across a healthy brain, a global OEF measurement may not be appropriate in some vascular pathologies (such as stroke), where local differences in OEF may be present, but with improved quality phase maps it will be possible to measure OEF more locally to the site of activation.

This work used a linear dependence of *R*_2_* on venous deoxygenation (β = 1) (Yablonskiy and Haacke, 1994) which assumes that the system is in the static dephasing regime (Kennan et al., 1994). This assumption is considered reasonable at 7 T, where larger frequency shifts occur around venules compared to at lower field strengths (Boxerman et al., 1995). The linear correlation observed between *R*_2_* and P_ET_CO_2_ and *R*_2_* and P_ET_O_2_ at 7 T (Driver et al., 2010; Croal et al., 2012a) is consistent with this assumption. This is an extravascular signal model, which is reasonable since the short T_2_* of venous blood at 7 T (Blockley et al., 2008) means that the intravascular GE signal contribution is small at TE = 25 ms (Duong et al., 2003). At the clinically accessible field strengths of 1.5 T and 3 T, the intravascular contribution to BOLD signal becomes significant and the extravascular BOLD signal includes a significant diffusive component, so the relationship between *R*_2_* and *Q* is no longer linear (Ogawa et al., 1993; Kennan et al., 1994; Boxerman et al., 1995). An extra compartment could be added to the BOLD signal model to account for the intravascular signal component, or this could be suppressed by using bipolar diffusion gradients. The supra-linear relationship, relating *R*_2_* and *Q* by the power β, widely used for low field calibrated BOLD (Davis et al., 1998) could be adopted here (e.g. β = 1.5 at 1.5 T).

Common to any hyperoxia-based calibration study, a possible source of error is a change in arterial blood oxygenation on hyperoxia, which will contribute to the hyperoxia BOLD signal. Considering the changes in arterial and venous saturations on hyperoxia illustrated in Fig. 1b, the effect of hyperoxia on venous blood susceptibility is about five times greater than the effect on arterial blood susceptibility. Specifically an increase in arterial oxygen saturation of 0.017 (for oxygen partial pressure PaO_2_ changing from 110 to 500 mm Hg) will cause a decrease in volume susceptibility (less paramagnetic, smaller shift relative to tissue) of Δ*χ*_*v*_ = − 0.0018 × 10^− 6^ (cgs units), based on Δ*χ*_*v*_(fully deoxygenated − fully oxygenated haemoglobin) = 0.264 × 10^− 6^ (Spees et al., 2001) and a haematocrit of Hct = 0.4. When this is combined with the fact that the venous blood volume is about three times greater than the arterial blood volume (Lee et al., 2001) this leads to approximately a 15 fold larger effect of hyperoxia on venous blood signal than on arterial blood signal. Assuming there is no change in arterial saturation on activation, then this will lead to a small constant overestimation of both M and M′ which will lead to a small underestimation of rvCBV and hence q_act_.

In contrast, hyperoxia will also lead to an increase in dissolved oxygen in arterial plasma which will cause an increase in arterial susceptibility (more paramagnetic, bigger shift relative to tissue). With the amount of dissolved oxygen determined by *ε* ⋅ PaO_2_ in Eq. (9) the volume susceptibility contribution of oxygen dissolved in blood plasma is given by:

(13) $$\chi_{v}\left({\text{PaO}}_{2}\right)=\frac{\chi_{m}}{V_{m}\times 10^{3}}\cdot \varepsilon \cdot {\text{PaO}}_{2}$$

where *ε* = 0.0031 ml/(dl_blood_·mm Hg) is the solubility coefficient of oxygen in blood, *χ*_*m*_ = + 3415 × 10^− 6^ cm^3^ mol^− 1^ (CRC, 2010) is the molar susceptibility of oxygen (cgs units) and  *V*_*m*_ = 24.5 L/mol is the molar volume, the volume occupied by 1 mol of ideal gas at room temperature and atmospheric pressure. Therefore the contribution of dissolved oxygen to blood susceptibility is *χ*_*v*_(PaO_2_ = 110 mm Hg) = 0.0005 × 10^− 6^ at normoxia and *χ*_*v*_(PaO_2_ = 500 mm Hg) = 0.0022 × 10^− 6^ at hyperoxia (cgs units). The resulting increase in susceptibility of Δ*χ*_*v*_ = + 0.0017 × 10^− 6^ will cause a small overestimation of rvCBV and q_act_, partially cancelling the underestimation of rvCBV and *q*_*act*_ predicted in the previous paragraph due to increased arterial haemoglobin saturation with hyperoxia. To put both these changes in arterial susceptibility in context, the estimated change in venous oxygenation saturation of 0.068 ± 0.003 due to hyperoxia and 0.14 ± 0.01 due to the motor task will correspond to a susceptibility change of 0.0072 × 10^− 6^ and 0.0148 × 10^− 6^ respectively (cgs units).

If the arterial blood volume changes on activation, this will also cause a change in signal on activation that is unaffected by hyperoxia, leading to an error in the estimate of q_act_ (but not in rvCBV). Considering the relative changes in the arterial and venous blood volumes, and the  T_2_* of arterial and venous blood and tissue, it is estimated that this will have an effect of about 5% on the difference in the intercepts in Fig. 1a that is used to estimate q_act_.

The model used here assumes that q_act_ is the same at both normoxia and hyperoxia which in turn assumes negligible changes in both CBF and CMRO_2_ on hyperoxia. The effect of hyperoxia on vasoconstriction and CBF is a matter of debate in the literature. Studies measuring CBF during hyperoxia have used fixed inspired gas mixtures to induce hyperoxia, and showed a decrease in CBF with hyperoxia (Kety and Schmidt, 1948; Watson et al., 2000; Kolbitsch et al., 2002; Bulte et al., 2007b). Attempts have been made to correct for this CBF decrease based on a look up table (Chiarelli et al., 2007a). However, as well as hyperoxia, these fixed inspired gas mixtures cause hypocapnia (reduced P_ET_CO_2_), which will cause a decrease in CBF. Graded hypercapnia has been used to try to separate hypocapnic from hyperoxic effects on CBF, measured using continuous-ASL (Floyd et al., 2003), finding a decrease in CBF with hyperoxia. However, in more recent work (Zaharchuk et al., 2008), the apparent decrease in CBF measured by continuous-ASL was mostly accounted for by a change in arterial blood T_1_ due to hyperoxia, rather than an actual CBF decrease. Recent work has found no change in global CBF on hyperoxia, when maintaining isocapnia during hyperoxia and measuring flow using phase contrast MRI (which is insensitive to T_1_ changes) and arterial CBV (Croal et al., 2012b). The effect of hyperoxia on CMRO_2_ has not been addressed in the literature and needs further investigation.

In this experiment, the change in P_ET_CO_2_ during hyperoxia was − 0.5 ± 0.1 mm Hg, less than that for an equivalent fixed inspired hyperoxia method (ΔP_ET_CO_2_≈ − 3 mm Hg during a 60% O_2_ challenge (Bulte et al., 2007b)). The effect on CBF and resulting BOLD signal is expected to be insignificant (~ 2% decrease for CBF (Noth et al., 2006) and ~ 0.2% decrease for BOLD (Driver et al., 2010)). However, in this study a brief overshoot in P_ET_CO_2_ of 1–2 mm Hg occurred during the transition to hyperoxia. In most cases this overshoot recovered back to baseline by the time of the motor task, but it did overlap with some motor trials, which were therefore discarded. It is likely that the RespirAct™ algorithm could be modified to minimise this effect. However, any method inducing hyperoxia whilst maintaining isocapnia would be suitable for this technique.

The susceptibility difference at the air–tissue interface in the oral cavity and frontal sinus will increase on hyperoxia, leading to increased field inhomogeneity across the brain (Blockley et al., 2009; Pilkinton et al., 2011), which will be worse at higher field strengths. Changing the oxygen fraction of air from 21 to 60% O_2_ changes the volume susceptibility of air by Δ*χ*_*v*_ = 0.054 × 10^− 6^ (cgs units, assuming *χ*_*m*_(O_2_) = + 3415 × 10^− 6^ cm^3^ mol^− 1^ and *χ*_*m*_(N_2_) = − 12 × 10^− 6^ cm^3^ mol^− 1^ (CRC, 2010)). For the EPI acquisition used here, the resulting change in field inhomogeneity has been shown to cause image distortion and intra-voxel dephasing close to the frontal sinus, but to have small effects in the motor cortex that would not significantly affect the results presented here (Driver et al., 2011).

## Conclusions

A new approach to BOLD calibration has been proposed and implemented, where a task is performed both at normoxia and hyperoxia. This uses hyperoxic BOLD contrast to estimate the change in venous blood oxygenation and venous CBV during a motor task. OEF was estimated from the phase data, allowing the effect of hyperoxia on venous blood oxygenation to be estimated on a subject by subject basis. By including an ASL measurement, the relative change in CMRO_2_ was estimated. The measured change in CMRO_2_ agrees with previous work, using other methods, but unlike those methods, this approach does not assume a poorly characterised relationship between vCBV and CBF.

## Figures and Tables

**Table 1:** Table of parameters.

Parameter	Description
Y	Blood oxygenation fraction (haemoglobin oxygen saturation fraction)
*Q*	Deoxygenated haemoglobin (dHb) fraction (= 1 − Y)
*Q*_0_	Normoxia, resting *Q*
Δ*Q*_h_	Absolute change in *Q* upon hyperoxia
q_h_	Relative change in *Q* upon hyperoxia (such that Δ*Q*_h_ = *Q*_0_·q_h_)
Δ*Q*_act_	Absolute change in *Q* due to the motor task
q_act_	Relative change in *Q* due to the motor task (such that Δ*Q*_act_ = *Q*_0_·q_act_)
CBF	Cerebral blood flow
rCBF	Relative change in CBF due to the motor task (rCBF = ΔCBF/CBF_0_)
CBV	Cerebral blood volume
vCBV	Venous cerebral blood volume
rvCBV	Relative change in vCBV due to the motor task (rvCBV = ΔvCBV/vCBV_0_)
CMRO_2_	Cerebral metabolic rate of oxygen consumption
rCMRO_2_	Relative change in CMRO_2_ due to the motor task (rCMRO_2_ = ΔCMRO_2_/CMRO_2,0_)
*α*	Coupling relationship between CBV and CBF (see Eq. (1))
β	Power law relationship relating extravascular transverse relaxation to Y
M	Calibration parameter, as used in previous BOLD calibration studies

**Table 2:** Individual subject results for the BOLD motor activation mask ROI, showing rvCBV, q_act_ and ΔY_act_ in response to the motor task.

Subject #	Paradigm	rvCBV (%)	q_act_ (%)	ΔY_act_
1	A	36.8	− 36.9	0.148
2	A	23.6	− 56.1	0.224
3	A	33.3	− 33.6	0.134
4	A	33.7	− 39.9	0.160
5	A	18.7	− 36.9	0.148
6	A	32.1	− 31.0	0.124
7	A	30.7	− 35.2	0.141
8	B	44.6	− 43.4	0.174
Mean ± SEM		32 ± 3	− 39 ± 2	0.157 ± 0.007

**Table 3:** Individual subject results for the ‘combined mask’, including results using both an assumed *Q*_0_ = 0.4 and results estimated from phase measurements of *Q*_0_.

Subject #	Paradigm	rCBF (%)	ΔY_act_	Assumed *Q*_0_ = 0.4:		Measured *Q*_0_:		
q_act_ (%)	rCMRO_2_ (%)	*Q*_0_	q_act_ (%)	rCMRO_2_ (%)
5	A	58.0	0.116	− 29.0	12.2	0.424	− 27.4	14.8
6	A	57.8	0.119	− 29.8	10.9	0.487	− 24.4	19.3
7	A	87.2	0.103	− 25.8	39.0	0.381	− 27.0	36.7
8	B	87.4	0.164	− 40.9	10.7	0.346	− 47.3	− 1.1
Mean ± SEM		73 ± 10	0.125 ± 0.015	− 31 ± 4	18 ± 8	0.410 ± 0.035	− 32 ± 6	17 ± 9

## Appendix A

Eq. (12), which allows *Q*_0_ to be estimated from the ratio of phase change between hyperoxia and normoxia and Δ*Q*_h_, is derived as follows. The assumption is made that on the unwrapped, filtered phase data, the phase shift in the region immediately around the sagittal sinus is dominated by the susceptibility of the blood in the sagittal sinus so

(A.1) $$a=\frac{\phi_{\text{HO}}}{\phi_{\text{NO}}}=\frac{\chi_{\text{HO}}}{\chi_{\text{NO}}}$$

where *χ* is the susceptibility of the sagittal sinus relative to tissue, and the subscripts denote the hyperoxia (HO) and normoxia (NO) conditions. This equation makes no assumptions about the spatial nature of the field shift around the vein, only that the field shift around the vein is dominated by the vein and is proportional to the deoxygenation of the vein. Assuming that blood plasma and tissue have the same susceptibility as water, the sagittal sinus susceptibility can be related to blood oxygenation (Y) by:

(A.2) $$\chi_{\text{NO}}=\text{Hct}\left(\text{Y}\cdot \text{Δ}\chi_{\text{oxy}}+\left(1-\text{Y}\right)\cdot \text{Δ}\chi_{\text{deoxy}}\right)$$

(A.3) $$\chi_{\text{HO}}=\text{Hct}\left(\left(\text{Y}+{\text{ΔY}}_{\text{h}}\right)\cdot \text{Δ}\chi_{\text{oxy}}+\left(1-\text{Y}-{\text{ΔY}}_{\text{h}}\right)\cdot \text{Δ}\chi_{\text{deoxy}}\right)$$

where Hct is the haematocrit fraction and ΔY_h_ = − Δ*Q*_h_. Eqs. (A.2) and (A.3) can be combined using Eq. (A.1), and be rearranged to give

(A.4) $$\text{Y}=\frac{\frac{-{\text{ΔY}}_{\text{h}}\cdot \left(\text{Δ}\chi_{\text{oxy}}-\text{Δ}\chi_{\text{deoxy}}\right)}{\left(1-a\right)}-\text{Δ}\chi_{\text{deoxy}}}{\left(\text{Δ}\chi_{\text{oxy}}-\text{Δ}\chi_{\text{deoxy}}\right)}\text{.}$$

Blood oxygenation fractions can be converted to dHb fractions by substituting Y = (1 − *Q*) and ΔY_h_ = − Δ*Q*_h_, giving Eq. (12).
